# Miscellanea

**Published:** 1856-04

**Authors:** 


### MISCELLANEA.

### PAUPERISM IN ENGLAND AND WALES.

On the 1st of January, 1855, 850,453 paupers received relief in England and Wales; and on the 1st of January of the present year, 876,655, out of a population calculated at 16,521,245, according to last census. This relief was distributed by six hundred and twenty-four unions. Of those relieved (in-door and out-door) on January 1st, 1855, 144,500, and on January 1st, 1856, 152,174 were able-bodied adults. On the day named in 1855, the in-door relief was supplied to 22,788 adult able-bodied persons of both sexes exclusive of vagrants; and out-door, to 121,712. On the same day 1856, in-door to 23,496, and out-door to 128,678. Amongst the relieved (in-door and out) on January 1st, 1856, 998 were married men, 1265 were married women, 164 were cases of urgent necessity, 18,526 were cases of sickness, 7579 were cases of sickness or accident of the family or for funerals, 4967 were on account of want of work, 25,595 were wives of adult males, 52,653 were widows, 5820 were single women without children, 3281 were mothers of illegitimates, 2182 were wives whose better (worse?) halves were in gaol, 2794 were wives of sailors, soldiers, or marines, and 5117 were wives of other non-resident males. The sum total of moneys expended in such relief for the half-year ending Michaelmas 1854 was £1,964,208; and for the same term ending Michaelmas 1855, £1,975,832. This expense was borne by 14,130 parishes. The expenditure, however, does not include parishes under Local Acts, Gilbert's Act, and the 43rd of Elizabeth, the total expenditure of which is stated at about £176,600.

In-maintenance consists of the cost of food and necessaries supplied in the workhouse: and in the six months ending Michaelmas 1855, it amounted to £466,490. Out-relief consists of relief in money or kind, or by way of loan to the out-door poor, it amounted in the same term to £1,509,412. The increase of expense in the six months ending Michaelmas 1855, was £29,624 over that of the same period in 1854.

---

### GRAIN AND FLOUR IMPORTED INTO IRELAND.

From the 1st day of January, 1855, to the 31st of Dec., 1855, there were imported into Ireland quantities of corn, grain, meal and flour from foreign countries and British possessions, amounting to 992,771 quarters; and from Great Britain 782,252 quarters. During the same period, there were imported into Great Britain from Ireland, 1,964,655 quarters of oats and oatmeal, 171,030 quarters of wheat and wheat-flour, 58,078 quarters of barley and barley-flour, 24,648 quarters of beans and bean-meal, 4954 quarters of malt, and 61,363 quarters of other forms of grain or meal; making a gross importation from Ireland to Great Britain of 2,226,650 quarters of eatable stuffs. If the good times are not come to the Emerald Isle now, could she feed her neighbours at this rate?

---

### HOMES FOR NEEDLEWOMEN.

It affords us great pleasure to be able to state that, through the benevolent exertions of the Viscountess Goderich and Lady Hartwell, a comfortable and respectable home for needlewomen has been established in Manchester Street, Manchester Square. The risk of the undertaking is, we believe, at present borne by these ladies. The house or home is presided over by a competent matron, is supplied with every convenience for cooking and sleeping, and with a capacious common sitting room. Medical attendance is kindly supplied by Dr. Edwards; and, by a strict economy, these advantages are brought within the limited means of the London sempstress, who is so fortunate as to have the opportunity of availing herself of them.

The precedent is excellent, and deserves an extended application in the metropolis. For this great purpose, however, organisation and concentration of purpose are required. The subject comes, indeed, within the sphere of municipal government; and that parish would be honoured that should produce a vestry knight, chivalrous enough to lead his colleagues to an earnest discussion on so important a point. He would be a *bonâ fide* legislator, too, such a man, not a mere talk forger; for he would strike at the root of a great social evil, and might confer immediate happiness, or comfort at least, on many hundred life-wearied innocents, who only become familiar with sin because they are wedded to it by misery. We shall watch the success of the undertaking of Ladies Goderich and Hartwell with great interest, with every desire to render such aid as it may be in our power to bestow, and with the hope that some of our influential readers may share in our devotion to so good a cause.

---

### POISONOUS EFFECTS OF TURPENTINE VAPOUR.

M. Marchal de Calvi related to the Academy of Sciences in Paris, on December 10th, the case of a woman who had lived for some days in a newly painted room. The first symptom which she experienced was colic; but soon she became prostrated, her face was deadly pale, the eyes sunken, the lips could scarcely be moved, the breath was cold, the voice was lost, the limbs were cold, the pulse almost imperceptible, the countenance anxious; the intellect, however, remained perfect, and the patient felt as if she were about to die. Under the use of external and internal stimulants she rallied, but did not perfectly recover for a month.

Some experiments made by M. Marchal, in conjunction with M. Mialhe, tend to shew that the lead is fixed in paint, and that therefore the poisonous symptoms produced by fresh paint cannot be attributed to it. From other experiments, M. Marchal concludes that the vapour of turpentine produces poisonous effects on man and animals. The conclusions at which the author arrives are: 1. White lead is fixed in paint, and is in no way concerned in the production of the poisonous symptoms arising from inhabiting a newly painted room. 2. These symptoms are due to the vapour of turpentine. 3. The danger is the same, whether the base of the paint be lead or zinc. 4. There is danger of poisoning by the turpentine as long as the paint is not perfectly dry; and it is safest not to inhabit a newly painted room until all smell has disappeared. 5. Poisoning by turpentine enters into the same category as poisoning by the emanations from flowers. 6. The emanations from flowers act in two ways—idiosyncratically, or as poisons. 7. The action of turpentine is chiefly depressing. 8. Energetic stimuli constitute the proper treatment; the peristaltic action of the bowels should be excited by appropriate means. The last two conclusions are not absolute, not being founded on a sufficient number of observations. (*Gazette Médicale de Paris,* Dec. 29, 1855.)

---

### POISONING BY SULPHURET OF CARBON AMONG WORKMEN IN INDIA-RUBBER MANUFACTORIES.

At the meeting of the Academy of Medicine on Jan. 15th, M. Delpech stated that he had arrived at the following conclusions with regard to the workmen in India-rubber manufactories.

1. That workmen in caoutchouc are liable to accidents which consist in (*a*) slight disturbance of digestion—loss of appetite, nausea, vomiting, diarrhœa, and constipation: (*b*) disturbance of the intellectual functions—hebetude, loss of memory, extreme restlessness, and unaccountable violence; (*c*) more serious disturbance of the nervous functions—cephalalgia, vertigo, disturbance of sight and hearing, impotence, and various forms of paralysis. 2. That observations made on men and experiments on animals, who become affected in the same way, lead to the conclusion that these symptoms are to be attributed to the inhalation of the vapour of sulphuret of carbon. (*Gazette Médicale de Paris*, Jan. 19th, 1856.)

---

### SANITARY APPLICATION OF CHARCOAL.

In a letter to the *Association Medical Journal*, Mr. G. M. Stansfeld suggests that square frames, made as light as possible, and covered with very fine wire netting, should be filled with *platinised charcoal*, and suspended at a convenient height from the ceiling in the wards of fever and general hospitals, or in the sick room in private houses.

The principle of suspension should be similar to the manner in which the punka is slung in Indian apartments; and by agitating the frames in a similar manner, most probably a more rapid decomposition and absorption of the impure air might be produced.

One, two, three, or more frames might be suspended, in proportion to the size of the ward; and a very simple contrivance would allow of their being moved to different positions in the apartments, as might be desired. The frames might be made of any size; but a medium size, framed as follows, would perhaps be best:—two slight frames, each three feet square, connected by four cross pieces, two inches long each; the intervening spaces to be filled in with fine wire netting, and the case then to be filled with small pieces of platinised charcoal. The frames should be suspended over the beds of the patients, and as low as possible, without being in the way of the attendants.

---

### MORTALITY IN THE CITIES OF THE UNITED STATES.

The number of deaths in New York city last year was 28,458, being 1 to every 21·95 of the population; in Philadelphia the number of deaths was 11,811, or 1 to every 42·33 of the population; in Baltimore 5,738, or 1 to every 36·54 of the population; in Boston 4,418, or 1 to every 36·21 of the population. In New York the ratio of deaths by consumption was 951; in Philadelphia 842; in Baltimore 615; in Boston 579.

